# Chemotypic Diversity, Antioxidant, Antimicrobial, and Antibiofilm Activities of Essential Oils from Arid-Grown *Ocimum basilicum* and *Ocimum africanum* and Their Representative Active Components

**DOI:** 10.3390/molecules31172940

**Published:** 2026-08-22

**Authors:** Samah Mechmechani, Abdullah A. Shaito, Mai M. Karousa, Maria Angelica M. Rocha, Diana C. G. A. Pinto, Mohammad Al-Khater, Moneer Ali, Mohamed M. Hassona, Layal Karam

**Affiliations:** 1 Department of Nutrition Sciences, College of Health Sciences, QU Health, Qatar University, Doha P.O. Box 2713, Qatar; 2 Biomedical Research Center (BRC), QU Health, Qatar University, Doha P.O. Box 2713, Qatar; 3 Biomedical Sciences Department, College of Health Sciences, QU Health, Qatar University, Doha P.O. Box 2713, Qatar; 4 College of Medicine, QU Health, Qatar University, Doha P.O. Box 2713, Qatar; 5 LAQV-REQUIMTE, Department of Chemistry, University of Aveiro, Campus de Santiago, 3810-193 Aveiro, Portugal; 6 Torba Farm, Doha P.O. Box 5825, Qatar; 7 Qur’anic Botanic Garden, Hamad Bin Khalifa University (HBKU), Education City, Doha P.O Box 1148, Qatar

**Keywords:** *Ocimum basilicum*, *Ocimum africanum*, Qatar, essential oils, eugenol, GC-MS, antibiofilm activity, antioxidant activity, food preservation

## Abstract

Plants grown in arid environments can produce distinct secondary metabolite profiles as adaptive stress responses. This study evaluated the antimicrobial, antioxidant, and antibiofilm activities of essential oils (EOs) from two *Ocimum* species cultivated in Qatar: *Ocimum basilicum* L. (OB-S, sweet basil) and *Ocimum africanum* Lour. (OA-L, lemon basil). GC-MS analysis revealed distinct chemotypes: OB-S was linalool-rich (31.53% linalool and 12.91% eucalyptol), while OA-L was citral-dominant (78.34% geranial/neral). OB-S exhibited superior biological activity, showing a lower minimum inhibitory concentration against *Listeria monocytogenes* (MIC = 5 mg/mL) than OA-L (MIC = 10 mg/mL). Consistent with its higher total phenolic content (999.06 ± 8.30 vs. 37.77 ± 3.25 µg GAE/g EO), OB-S demonstrated significantly greater antioxidant capacity (DPPH EC_50_ of 162.69 ± 0.78 vs. 939.38 ± 0.68 µg/mL). Further evaluation of OB-S EO and its major constituents (linalool, eugenol, estragole, eucalyptol) against *L. monocytogenes* biofilms revealed that while the whole EO reduced biofilm viability at 2MIC, isolated phytocompounds were more potent. Eugenol achieved the greatest reduction (reaching up to 5 log CFU/cm^2^), suggesting that it may be a major contributor to OB-S EO’s antibiofilm activity. These findings highlight the distinct chemotypic and bioactive profiles of these *Ocimum* species cultivated under arid conditions while also demonstrating eugenol’s potential for food-safety applications as a natural food preservative.

## 1. Introduction

Pathogenic microorganisms and their resilient biofilms present significant health and economic challenges across multiple domains, including clinical settings, pharmaceuticals, and food industry, where they compromise both safety and operational integrity [1,2,3]. Foodborne such as *Salmonella enterica*, *Pseudomonas aeruginosa*, and *L. monocytogenes* can persist as resilient surface biofilms, enabling them to withstand extreme desiccation and survive for extended periods [4]. Encased within protective extracellular polymeric substance (EPS) matrices, these bacteria exhibit markedly enhanced tolerance to environmental stress, commercial cleaning agents, and standard sanitation procedures, thereby posing severe challenges to food hygiene and public health [5]. This biofilm mode of growth further exacerbates the crisis of antimicrobial resistance (AMR). Bacteria within biofilms can display resistance levels up to 1000 times higher than their free-floating planktonic counterparts, which severely limits the efficacy of conventional disinfectants [6,7,8]. Furthermore, the extensive and often indiscriminate application of synthetic chemical sanitizers in food systems has accelerated the emergence of resistant strains [9]. This growing resistance threatens the long-term efficacy of current pathogen control strategies, highlighting an urgent need for alternative, sustainable, and natural antimicrobial interventions capable of targeting both planktonic cells and biofilm matrices.

In this context, plant-derived essential oils (EOs) are gaining considerable attention as promising natural alternatives for industrial applications in the food sector. EOs are widely recognized for their broad-spectrum antimicrobial activity and their ability to disrupt and eradicate established biofilms on food-contact surfaces [10,11,12]. Crucially, many EOs also possess potent antioxidant properties, making them attractive dual-action candidates for natural food preservation. Beyond preserving food quality, these bioactive essential oils offer extensive health benefits to consumers by lowering systemic oxidative stress, replacing potentially harmful synthetic preservatives, and demonstrating potent anti-inflammatory, immune-modulating, and chronic disease-preventive properties [13,14,15,16]. The combined antimicrobial and antioxidant functionality of EOs positions phytochemicals as viable, clean-label substitutes for synthetic preservatives. In addition to their antimicrobial and antioxidant properties, essential oils from *Ocimum* species have attracted considerable interest as natural pest-management agents [17]. Previous studies have demonstrated the insecticidal, fumigant and repellent activities of *O. basilicum* essential oil and its major constituents against several agricultural and stored-product insect pests [18,19,20]. This broad spectrum of biological activities further highlights the multifunctional value of *Ocimum* essential oils and supports continued investigation of their potential applications in sustainable food protection systems [21].

The biosynthesis and efficacy of these EOs are profoundly shaped by the interaction between the plant’s genetic print and its cultivation environment. Climate change and increasing environmental pressures, especially in arid and semi-arid regions, significantly influence plant secondary metabolism [22]. While the distinct chemical profiles (chemotypes) of EOs are fundamentally determined by their unique genetic characteristics, abiotic stressors typical of arid environments, such as high temperatures, intense UV radiation, and limited water availability, strongly influence their physiological and metabolic responses [23]. These factors upregulate the plant’s secondary metabolic pathways, prompting the accumulation of protective volatile compounds (like terpenoids and phenolics) to safeguard cellular integrity [24]. Among the most commercially and medicinally important aromatic plants, species of the genus *Ocimum* (basil) are valued worldwide for their culinary uses and rich phytochemical profiles [25,26]. *Ocimum basilicum* L. (sweet basil) is globally recognized for its sweet, floral aroma, dominated by linalool and eugenol, while *Ocimum africanum* Lour. (lemon basil), a distinct species often used in traditional medicine and cuisine, is characterized by a fresh, citrus-like scent due to its high citral content [27,28]. The pronounced differences in their chemical profile and their adaptability to diverse climates make them ideal candidates for investigating how environmental stress shapes their functional properties, as variations in temperature, water availability, and solar radiation are known to dictate their EO yield, chemotypic profile, and subsequent biological activity [29,30]. Crucially, the EOs derived from these plants contain a diverse profile of volatile phytocompounds, including linalool, eugenol, methyl cinnamate, estragole, and citral, recognized for targeted antimicrobial and antioxidant mechanisms [25,31]. These active compounds exert their effects by penetrating bacterial cell membranes, inducing intracellular leakage, inhibiting quorum-sensing pathways, and utilizing phenolic hydroxyl groups to neutralize oxidative free radicals [14,32,33].

Despite these well-documented attributes, research has often remained fragmented, focusing either on antioxidant or planktonic antimicrobial activity, typically using plants cultivated in controlled, temperate climates. Consequently, there is limited understanding of *Ocimum* species grown in harsh, arid conditions, particularly regarding the functional performance of their EOs and active components against biofilm-forming food pathogens. Therefore, this study aimed to characterize and compare the antioxidant and antibiofilm activities of EOs from two distinct *Ocimum* species, *O. basilicum* L. and *O. africanum* Lour., cultivated in the arid environment of Qatar. We investigated the chemical profiles of the EOs, evaluated their antimicrobial efficacy against the foodborne pathogen *L. monocytogenes*, and assessed the targeted antibiofilm eradication efficacy of the most potent EO and its key active components. This work seeks to leverage the properties of these arid-grown *Ocimum* chemotypes, which represent valuable models for studying potential stress-induced secondary metabolites, for potential application as sustainable, multi-functional solutions in modern food-safety systems.

## 2. Results and Discussion

### 2.1. Essential Oil Yield

The extraction process yielded approximately 8 g of yellow-colored basil essential oil for each species, corresponding to a recovery rate of approximatively 0.02% (*w*/*w*) for both OB-S and OA-L. These values are substantially lower than those commonly reported in the literature for *Ocimum* species cultivated in more temperate or optimized environments. For instance, Pirmoradi et al. [34] reported EO yields ranging from 0.6% to 1.1% (*w*/*w*) for 21 accessions of *O. basilicum* L. cultivated in Iran. Similarly, Ozcan and Chalchat [35] found yields of 1.25% from air-dried aerial parts of *O. basilicum* grown in Turkey using hydrodistillation. Rashid et al. [36] reported yields between 0.40% and 0.54% (*w*/*w*) for hydro-distilled sweet basil flower EO across different regions. In addition, Hussain et al. [30] studied the EO yield of *O. basilicum* across different weather conditions. They found that the hydro-distilled EO content ranged from 0.5% to 0.8%. A relatively low essential oil yield was observed in the studied samples, which were cultivated under the arid environmental conditions of Qatar. Severe environmental factors, such as particularly intense irradiance, high temperatures, and water scarcity, have been shown to negatively impact the morphological traits (e.g., leaf size, glandular trichome density) and overall biomass of basil, consequently reducing the net essential oil yield [37]. Furthermore, prolonged exposure to extreme heat can induce the premature volatilization of low-molecular-weight aromatic compounds prior to harvest, significantly decreasing the final extractable essential oil content [29,38,39].

### 2.2. Basil Essential Oil Chemical Composition

**Figure 1:**
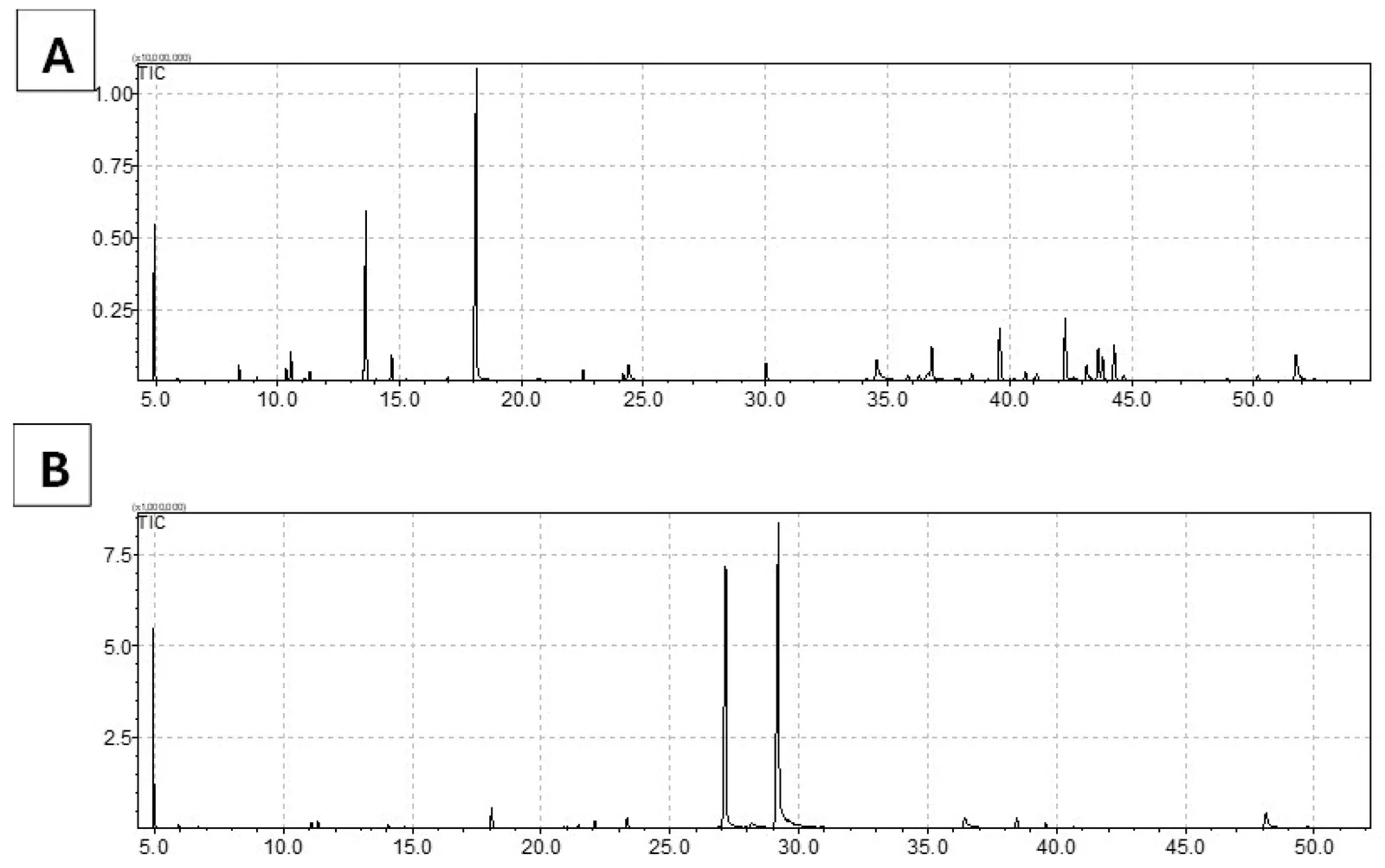
Total ion chromatogram (TIC) of essential oils of (**A**) *Ocimum basilicum* L. and (**B**) *Ocimum africanum* Lour.

Gas chromatography–mass spectrometry (GC-MS) analysis resolved 20 and 8 constituents in the EOs of OB-S and OA-L, respectively (Table 1; Figure 1). The comparative chemical profiling revealed a profound compositional divergence between the two species. The EO of OB-S exhibited a classic commercial profile characterized by a high abundance of linalool (31.53%) and 1,8-cineole (eucalyptol, 12.91%), alongside 4-hydroxy-4-methylpentan-2-one (7.47%), germacrene D (6.42%), and -bergamotene (6.11%), τ-Cadinol 3.91%, γ-Cadinene (3.86%), α-Bulnesene (3.39%) and eugenol (3.17%). This linalool/1,8-cineole-dominant chemotype is characteristic of commercially species of *O. basilicum*, which synthesize the sweet, floral, and herbaceous volatile notes selected historically for culinary and industrial applications [25,40]. For instance, the observed dominance of linalool (31.53%) in our *O. basilicum* L. sample aligns closely with profiles documented across several warm-temperate, Mediterranean, and arid climates. This trend is reflected in the *O. basilicum* basil essential oils from Brazil (78.12%) [41], Croatia (28.6%) [42], Serbia (58.6–30.3%) [40], Tunisia (40.1–7.65%) [43], Egypt (48.4–43.5%) [44], Oman (69.92%) [45], and Colombia (41.1–37.9%) [46]. These levels suggest that elevated temperatures and high solar radiation actively stimulate monoterpene accumulation. Conversely, lower linalool levels reported in Malaysia (1.02%) [47] and Slovakia (<0.05%) [48] suggest that cooler temperate conditions, or distinct soil compositions exert a suppressive effect on linalool biosynthesis.

In contrast, the taxonomically distinct OA-L EO displayed a specialized, citral-rich chemotype dominated almost exclusively by the geometric isomers geranial [(E)-citral, 45.33%] and neral [(Z)-citral, 33.01%], alongside 4-hydroxy-4-methylpentan-2-one (10.92%). This citral-dominant profile consistently align with reports from regions characterized by high temperatures and intense solar irradiance like *O. africanum* species from tropical Brazil (68.6–85.8%) [27], subtropical India (68.0–76.05%) [49], and the Mediterranean Çukurova region of Turkey (up to 89.28%) [50]. Similarly, robust citral levels in lemon basil are sustained under the wet-and-dry monsoonal climate of Thailand (50.2–54.8%) [51], reinforcing that warm environments optimize the enzymatic oxidation of monoterpene alcohols into their corresponding aldehydes. Conversely, markedly lower citral concentrations (6.49–17.37%) in *O. africanum* were reported in temperate regions like Poland [52] suggesting that cooler, lower-light conditions suppress these specific biosynthetic pathways, potentially diverting secondary metabolism toward alternative volatile constituents. In typical *O. basilicum* L. species, citral is generally absent or restricted to minor trace amounts, as their metabolic networks favor the biosynthetic pathways of monoterpene alcohols (e.g., linalool) or phenylpropanoids (e.g., estragole, methyl eugenol) [28]. The pronounced accumulation of neral and geranial in OA-L EO indicates a distinct hybridization background, typically arising from natural or selective crosses between *O. basilicum* and *O. africanum* [53]. Biochemically, this metabolic shift suggests a divergent enzymatic channeling of geranyl pyrophosphate (GPP). In these citral-rich hybrids, geraniol dehydrogenase actively oxidizes geraniol to citral isomers, redirecting carbon flux away from the linalool synthase pathway. Renowned for its crisp, lemon-like aromatic profile, this chemotype is valued in both food science and traditional culinary applications across Mediterranean and Asian regions [27].

Crucially, although these distinct volatile profiles are deeply mediated by genetic predispositions, their pronounced accumulation is significantly modulated by the extreme environmental conditions of their cultivation. Abiotic stressors inherent to arid climates, such as high thermal loads, intense solar irradiance, and water deficits, act as catalysts that alter the expression of terpene and phenylpropene biosynthetic enzymes and trigger robust plant defense mechanisms [26,54]. Under these suboptimal, stressful conditions, the plant actively upregulates its secondary metabolism to combat severe cellular oxidative damage [55]. This adaptive response not only increases the concentration and complexity of major essential oil constituents like linalool, eugenol, and citral [29,56,57] but it can also stimulate a marked accumulation of total phenolics and flavonoids [55,58,59]. Ultimately, the final phytochemical quality, essential oil yield, and potent antioxidant profiles of OB-S and OA-L represent a synergistic interaction between their inherent genetic chemotypes and their active metabolic adaptations to survive harsh environmental stress.

### 2.3. Basil Essential Oils Screening: Antimicrobial and Antioxidant Activities

#### 2.3.1. Antimicrobial Efficacy: Minimum Inhibitory Concentration Against *L. monocytogenes*

To assess the antimicrobial efficacy of the EOs obtained from the two *Ocimum* species, the MIC values were determined against the foodborne pathogen *L. monocytogenes* (Table 2). The MIC values for the OB-S and OA-L EOs were found to be 5 mg/mL and 10 mg/mL, respectively. Notably, the OB-S EO demonstrated the highest antimicrobial efficacy. This enhanced bioactivity is directly attributable to its complex GC-MS chemical profile. While the OA-L EO displays a restricted chemical profile comprising only 8 distinct constituents, the OB-S EO represents a diverse phytocomplex containing 20 distinct active compounds (Table 1). This chemical richness provides a diverse spectrum of monoterpenes and phenylpropanoids that may act synergistically or exhibit additive effects, enhancing the overall functional potential of the essential oil. The OB-S EO is particularly rich in oxygenated monoterpenes, specifically linalool (31.53%) and 1,8-cineole (eucalyptol, 12.91%), which are well-documented to act as potent membrane permeabilizers. Additionally, it contains eugenol (3.17%) and estragole (1.82%), phenylpropanoids renowned for their ability of the disruption of *Listeria* cell envelope [60,61]. Conversely, the antimicrobial action of the OA-L EO (MIC = 10 mg/mL) is likely associated with its electrophilic monoterpene aldehyde profile, which is dominated by the geometric isomers geranial [(E)-citral, 45.33%] and neral [(Z)-citral, 33.01%]. These isomeric aldehydes possess reactive carbonyl groups capable of participating in nucleophilic addition reactions (specifically Schiff-base formations) with the free amino groups of bacterial cell envelope proteins and membrane-bound enzymes [62]. This cross-linking of vital structural and metabolic proteins can compromise cell wall integrity, leading to severe membrane destabilization and irreversible cellular damage [63].

#### 2.3.2. Total Phenolic Content (TPC) and Radical Scavenging Capacity of the Ocimum EOs

The comparative analysis of the antioxidant radical scavenging potential and the total phenolic content (TPC) of the EOs revealed profound quantitative disparities between the two basil species. Specifically, the OB-S EO exhibited a prominent antioxidant capacity, with a decent EC_50_ of 162.69 ± 0.78 µg/mL. In contrast, linalool, estragole, and eucalyptol exhibited limited antioxidant activity [64] and did not achieve 50% radical scavenging at the highest experimentally tested concentration of 1000 µg/mL. The EC_50_ values estimated from the fitted dose–response curves were 3655.3 ± 2.01 µg/mL for linalool, 4860.7 ± 1.19 µg/mL for estragole, and 4974.09 ± 1.55 µg/mL for eucalyptol. These values exceed the tested range and should therefore be interpreted as extrapolated estimates (Table 3). These free-radical scavenging capacities correlated directly with the TPC of the respective EOs. The OB-S EO contained a remarkably high phenolic concentration of 999.06 ± 8.30 µg GAE/g of EO, outperforming the OA-L EO (37.77 ± 3.25 µg GAE/g of EO). This divergence in TPC and antioxidant efficacy is consistent with the differences in chemical complexity of the two EOs as demonstrated by GC-MS. The OB-S EO represents a diverse phytocomplex of 20 compounds, including the allylbenzene eugenol (3.17%). Eugenol is a phenolic compound whose free allylic hydroxyl group serves as hydrogen-atom donor, efficiently neutralizing DPPH free radicals via single-electron transfer and increasing TPC spectrophotometric readouts [65]. In contrast, the weak antioxidant capacities observed for the OA-L EO are biochemically attributed to its restrictive, non-phenolic profile. The volatile profile of OA-L is restricted to only 8 compounds and is dominated almost exclusively by aliphatic monoterpene aldehydes (citral isomers). Unlike phenolic compounds, these aldehydes lack the critical free hydroxyl groups required for robust hydrogen atom transfer or single electron transfer, resulting in a significantly lower thermodynamic capacity to stabilize free radicals [28,66].

In summary, these preliminary screening results confirmed that the OB-S EO possesses an enhanced antioxidant profile and antimicrobial activity against *L. monocytogenes*. Consequently, this EO, alongside four of its key volatile constituents, linalool, eugenol, eucalyptol, and estragole, was selected for further bioactivity evaluation. The selection of these isolated active components is further supported by their well-established antimicrobial activity and radical-scavenging mechanisms in previous studies [42,60,67,68,69,70,71,72,73].

### 2.4. Antimicrobial and Antioxidant Activities of OB-S EO and Its Selected Active Components

The MICs of the selected potential active constituents from OB-S EO (linalool, eucalyptol, eugenol, and estragole) were evaluated against the foodborne pathogen *L. monocytogenes* (Table 2), revealing MIC values of 10 mg/mL for eugenol, 20 mg/mL for linalool, and 40 mg/mL for both eucalyptol (1,8-cineole) and estragole (4-allylanisole). Among these, eugenol exhibited the highest bactericidal potency, a characteristic well documented in the literature [74,75,76,77], and was primarily driven by its ability to alter cell membrane permeability and disrupt transmembrane electrochemical gradients, which leads to the rapid leakage of intracellular adenosine triphosphate (ATP), proteins, nucleic acids, and diagnostic enzymes such as β-galactosidase and alkaline phosphatase [77]. This membrane-disrupting pathway is further validated by the previous work of Kim et al. [78], who were among the first to demonstrate the broad-spectrum antimicrobial potential of isolated volatile constituents like linalool and eugenol against key foodborne pathogens, including *Escherichia coli*, *S. enterica* serovar Typhimurium, *L. monocytogenes*, and *Vibrio vulnificus*. Furthermore, Zengin and Baysal [79] reported that terpene compounds, such as linalool, α-terpineol, and eucalyptol, induce severe physical damage to the cell envelope and irreversibly alter the morphological structure of *Staphylococcus aureus*, *S. enterica* serovar Typhimurium, and *E. coli* O157:H7. The biochemical explanation for this structural disruption may lie in the insertion of these hydrophobic terpenes into phospholipid bilayers and their interactions with membrane-anchored proteins, leading to cellular respiratory chain inhibition, the interruption of oxidative phosphorylation, the disruption of nucleic acid synthesis, and a loss of essential metabolic intermediates [80]. Furthermore, comparative studies by Auezova et al. [81] and Gharib et al. [82] on the cell envelopes of *E. coli* and *S. epidermidis* revealed that the distinct modes of action of allylic phenylpropenes (such as eugenol and isoeugenol) and propenylic phenylpropenes (such as estragole and anethole) are governed by their physical-chemical properties. Specifically, estragole and anethole demonstrated a specialized capacity to partition into and penetrate the outer membrane of Gram-negative bacteria, displaying an enhanced membrane-permeabilizing potency that is conferred by their lipophilic nature in comparison to their more polar counterparts, eugenol and isoeugenol [83].

Compared with these isolated components, the OB-S EO in this study exhibited a better MIC of 5 mg/mL, indicating that the whole EO possessed enhanced bactericidal activity against *L. monocytogenes* planktonic cells relative to some of its individual active components. This phenomenon is well-supported by previous research indicating that such enhanced bioactivity is due to the possible synergistic or additive interactions among the complex constituent matrix within the crude essential oil, which can work cooperatively to outperform the efficacy of isolated single molecules [84,85,86,87,88,89,90,91]. Conversely, some studies have demonstrated that specific isolated active components displayed equal or enhanced antimicrobial activity compared to their whole EOs [6,92,93,94]. For example, despite containing a substantial concentration of linalool, crude pummelo peel EO failed to inhibit *E. coli*, a pathogen that proved susceptible only to the pure, isolated linalool fraction [95,96]. In such scenarios, the reduced efficacy of the whole EO is often attributed to antagonistic interactions or dilutive effects among constituents that collectively dampen overall bioactivity [67,97,98].

Regarding the antioxidant profiling, the radical scavenging capacities of the selected isolated components, linalool, eucalyptol, eugenol, and estragole, were individually assessed via the DPPH assay (Table 3), revealing a profound quantitative disparity in antioxidant efficacy among the tested compounds. Eugenol exhibited enhanced scavenging capacity, with an EC_50_ of 5.31 ± 0.67 µg/mL, outperforming the ascorbic acid reference standard (EC_50_ = 23.91 ± 0.3 µg/mL). In contrast, the other major constituents (linalool, estragole, and eucalyptol) displayed minimal antioxidant activity exceeding 1000 µg/mL. These findings suggest that eugenol may be a main contributor to the enhanced radical scavenging activity of the whole OB-S EO (EC_50_ = 162.69 µg/mL). Although eugenol accounts for only a minor fraction (3.17%) of the sweet basil oil, its ability to donate hydrogen atoms via its phenolic hydroxyl group makes it a disproportionately strong radical scavenger. This dominant contribution of eugenol is consistent with existing literature. The potent, isolated antioxidant capacity of eugenol has been extensively documented in previous pharmacological studies [99,100]. For instance, Pripdeevech et al. [101] found that a linalool/eugenol chemotype of *O. basilicum* from Thailand displayed remarkable DPPH radical scavenging activity (IC_50_ = 26.53 µg/mL), suggesting that the observed antioxidant efficacy was largely attributable to its phenolic components. Similarly, Politeo et al. [42] determined that the antioxidant capacity (IC_50_ = 1.4 g/L) of an Austrian basil EO was mainly attributed to its eugenol content (5.9%), noting minimal antioxidant contributions from the more abundant monoterpenes. This was further corroborated by Lee et al. [102], who identified eugenol as the primary, dominant contributor to the antioxidant activity of basil volatile extracts from the USA, vastly surpassing the effects of major constituents like linalool or eucalyptol. Ultimately, these data support the conclusion that although eugenol is a minor active component by volume in the analyzed Qatari *O. basilicum* L. extract, its high individual phenolic character and antioxidant capacity may drive the EO’s overall antioxidant capacity. Beyond the observed antimicrobial and antioxidant effects, *Ocimum* essential oils exhibit a broad-spectrum bioactivity that extends to diverse applications. This multifunctional profile highlights the potential of *O. basilicum* essential oil as a health promoter and a natural agent for preserving food quality and safety, addressing both microbial spoilage and pest-related challenges [18,19,20].

### 2.5. Antibiofilm Activity of Ocimum basilicum L. Essential Oil and Its Selected Active Components

**Figure 2:**
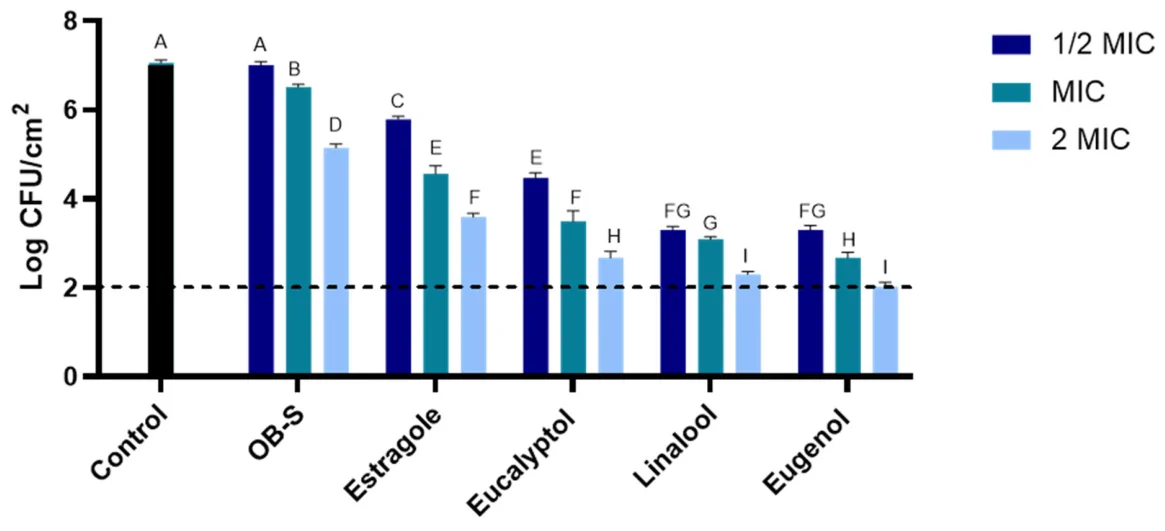
The effect of *Ocimum basilicum* L. (OB-S) essential oil and its active components at ½ MIC, 1 MIC and 2 MIC on the total viable biofilm count of *L. monocytogenes*. The black bar represents the control biofilms treated with phosphate buffer containing DMSO without any antimicrobials. Results are presented as the mean (±SD) of two independent experiments. The control group consisted of biofilms treated with tryptone salt broth containing DMSO. Different letters above the error bars (A, B, C, etc.) indicate significant differences (*p* < 0.05), while the same letters denote non-significant differences (*p* > 0.05). The detection limit for this method is 2 log CFU/cm^2^, represented by the dashed line.

Despite its effective performance in planktonic assays, a different dynamic emerged when evaluating the antibiofilm activity of the OB-S EO versus its selected active components (Figure 2). Biofilms of *L. monocytogenes* were grown on stainless steel for 24 h at 37 °C, yielding a bacterial count of approximately 7 log CFU/cm^2^. When treated with various concentrations (½ MIC, 1 MIC and 2 MIC), our results indicated that the ½ MIC and 1 MIC treatments for 15 min of the whole EO failed to significantly reduce the viable biofilm count. This reduced efficacy may be attributed to the physical and chemical complexity of the whole EO. Consequently, while these components may act additively or synergistically in planktonic suspensions, the multi-component nature of the EO may lead to physical hindrance, ultimately restricting the diffusion of active molecules through the protective extracellular polymeric substance (EPS) of the mature biofilm [103]. Disruption only became evident at higher concentrations, at 2 MIC resulting in a viable biofilm count reduction of about 2 log CFU/cm^2^ (*p* < 0.05). These findings align with the observations of Nazir et al. [104] who evaluated the efficacy of *O. basilicum* essential oil (EO) against *S. aureus* and *E. coli*. They similarly reported concentration-dependent inhibition, noting that maximum biofilm reduction was achieved only at elevated fractions of the EO. The antibiofilm potential of *O. basilicum* is documented in the broader literature [105,106,107], though it remains dependent on the applied concentration and the capacity of its constituents to penetrate the extracellular polymeric substance (EPS) matrix.

In contrast to the whole EO, the selected active components demonstrated higher antibiofilm efficacy, achieving significant log reductions compared to the control even at sub-inhibitory concentrations (*p* < 0.05). Eugenol (present at 3.17% in OB-S) demonstrated potent antibiofilm activity, yielding a 3.8 log CFU/cm^2^ reduction (*p* < 0.05) at just ½ MIC, which increased to 4.4 log CFU/cm^2^ (*p* < 0.05) at 1 MIC and peaked at 5 log CFU/cm^2^ (*p* < 0.05) at 2 MIC. Similarly, eugenol has consistently demonstrated potent antibiofilm activity against various challenging strains, including *S. aureus* and MRSA, even at sub-inhibitory concentration [32,68]. Additionally, eugenol exhibits anti-quorum sensing effects on *P. aeruginosa* [33]. Pérez-Conesa et al. [108] demonstrated that encapsulating eugenol in nonionic surfactant micelles effectively inactivates mature (48 h) *L. monocytogenes* biofilms, achieving rapid bacterial eradication within the first 10 min of treatment. These nonionic micelles bypass the biofilm’s defensive polymer matrix, diffusing through its porous structure to deliver the eugenol directly to embedded bacteria. Once delivered, the eugenol attacks the bacterial cells by disrupting the cell membrane, which depletes intracellular ATP and inhibits essential membrane-bound ATPase enzymes responsible for energy production and pH regulation [108]. This combined action of efficient delivery and targeted cellular attack leads to rapid bacterial cell death, transforming a viable biofilm into one composed of non-respiring cells within minutes [109,110]. Linalool followed closely, achieving an identical initial reduction of 3.8 log CFU/cm^2^ at ½ MIC which significantly increased to 4.0 log CFU/cm^2^ at 1 MIC, and reached a maximum reduction of 4.7 log CFU/cm^2^ at 2 MIC (*p* < 0.05). Previous studies demonstrated the high antibiofilm activity of linalool. Praseetha et al. [8] reported a concentration-dependent inhibition of *Streptococcus pyogenes* biofilms, initiating a 10% reduction at a concentration of 0.002% (*v*/*v*) and achieving a maximum of 91% disruption at 0.004% (*v*/*v*). Prakash et al. [111] further demonstrated that linalool’s antibiofilm efficacy is enhanced when formulated as nanoemulsions, resulting in 64.4% inhibition compared to 56.9% with pure linalool. For instance, Dias et al. [112] demonstrated that linalool’s antibiofilm activity against *L. monocytogenes* stems from its ability to suppress essential virulence factors, specifically by inhibiting cellular adhesion, quorum sensing, and motility, which are all crucial for the successful establishment and persistence of a biofilm. In addition, Lahiri et al. [70] found that eugenol and linalool directly influenced the synthesis of quorum-sensing proteins along with virulence factors, significantly hampering biofilm formation of *P. aeruginosa*.

For eucalyptol antibiofilm activity, it showed 2.6 log reduction at ½ MIC and reached a maximum 4.4 log reduction at 2 MIC. Followed by estragole that exhibited the least effective antibiofilm activity among the tested compounds, initiating at a 1.3 log reduction at ½ MIC and reaching a maximum of 3.5 log reduction at 2 MIC. Several research highlighted the efficacy of eucalyptol (1,8-cineole) and estragole (4-allylanisole) against biofilms. Wang et al. [113] found that 1,8-cineole inhibited *Escherichia coli* biofilm formation by 61.7%, 47%, and 35.1% at ½ MIC, ¼ MIC, and 1/8 MIC, respectively. Similarly, Merghni et al. [114] demonstrated that 1,8-cineole exhibited anti-adhesion properties against biofilm-forming MRSA strains, resulting in the eradication of pre-formed biofilms with reductions ranging from 77.46% to 90.81% at various concentrations. Additionally, Wongsariya et al. [115] reported that methyl chavicol achieved over 90% inhibition of *S. mutans* biofilms at concentrations between ¼ MIC and 1 MIC, while completely eliminating *S. sobrinus* biofilms at its MIC.

While the complex mixture of OB-S EO (MIC = 5 mg/mL) did not significantly reduce the viable biofilm count of *L. monocytogenes* at ½ MIC and 1 MIC, its selected active components (eucalyptol, estragole, linalool, and eugenol with individual MICs ranging from 10 to 40 mg/mL) demonstrated substantial antibiofilm efficacy at these levels. When tested relative to their respective MICs, the greater absolute mass of the active components seems to apply a significantly larger physicochemical force against the structural barriers of the biofilm, which drive enhanced reduction in biofilm-associated viable cells. However, against planktonic cells, the whole EO may rely on potential synergistic relationship: major constituents like linalool seem to operate at lower concentrations to impair motility, prevent initial attachment, and weaken cellular defenses, while trace active phenols like eugenol may contribute to membrane permeabilization and EPS matrix disruption. This complex multi-target mechanism may explain why OB-S remains effective as a broad-spectrum antimicrobial agent against planktonic bacteria. Conversely, overcoming the dense, protective matrix of an established biofilm requires a different dynamic. These complex structures appear to be more vulnerable to the targeted action of selected phytocompounds like eugenol and linalool. These findings, where selected active constituents outperform their whole EO antibiofilm activity, is widely demonstrated in recent literature. Pontanayodsakorn et al. [116] tested the antibiofilm activity of kaffir lime EOs from leaves (KLL) and peels (KLP) along with their active components, β-citronellol and terpinen-4-ol, which were the third most abundant compounds in KLL and KLP, respectively. The results indicated that the potency order for biofilm eradication at the MIC level was as follows: terpinen-4-ol (67.50%) > β-citronellol (58.38%) > KLL (35.38%) > KLP (17.45%). The maximum post-biofilm inhibition of KLL and KLP was less than 66.10% at concentrations exceeding their MIC levels [116]. In contrast, β-citronellol and terpinen-4-ol achieved up to 90% biofilm eradication at concentrations higher than their MIC values. Terpinen-4-ol also demonstrated over 90% pre-biofilm inhibition at its MICs, while KLP showed approximately 27.73% inhibition at the same level. Similar findings were reported by Nostro et al. [6] who demonstrated that carvacrol and thymol were more effective than oregano EO in inhibiting *S. aureus* and *S. epidermidis* biofilm formation in polystyrene microplates.

## 3. Materials and Methods

### 3.1. Plants Collection

Fresh aerial parts (leaves and stems) of *O. basilicum* L. (OB-S) were sourced from the Qur’anic Botanic Garden (QBG), a BGCI-accredited botanical garden and a member of Hamad Bin Khalifa University (HBKU), Qatar Foundation, located within Education City, Doha, Qatar (Accession No. 2010.28.03.6.00). In addition, the aerial parts of *O. africanum* Lour. (OA-L) were collected from Torba Farm, Al khor, Doha, Qatar (Accession No. 25°34′41.2″ N 51°18′41.4″ E). The plant specimens were formally identified and authenticated by botanical experts at each location. The plant materials were collected during the autumn harvesting season between October and November 2025. Both plant species were harvested at their peak developmental phases, specifically ranging from the late vegetative to full-flowering stages, which represent the optimal periods for essential oil accumulation. For each species, the aerial materials were gathered by cutting the stems at a height ranging from 10 to 15 cm above ground level, ensuring that the root systems were entirely excluded from the collection. To ensure a highly representative and genetically diverse chemical profile, sampling was performed across a large, healthy population of plants. For each species, a minimum of 100 individual plants were sampled. The collected materials from these individual plants were subsequently combined to form a single, homogeneous pooled (composite) sample for each species before essential oil extraction. Following collection, the fresh plant materials were immediately processed for EO extraction.

### 3.2. Antimicrobials and Reagents

Four active components were sourced from Sigma-Aldrich (St. Louis, MO, USA): linalool (≥97%, CAS number 78-70-6), eugenol (≥98%, CAS number 97-53-0), estragole (4-allylanisole, ≤100%, CAS number 140-67-0), and eucalyptol (1.8 cineole, ≥99%, CAS number 470-82-6). MgSO_4_ was sourced from Sigma-Aldrich, Doha, Qatar, Hexane from Sigma-Aldrich, Sintra, Portugal, and Dey–Engley Neutralizing Broth Base from HIMEDIA, Mumbai, India. Dimethyl sulfoxide (DMSO; Sigma-Aldrich, Saint-Quentin-Fallavier, France) at a final concentration of 2% (*v*/*v*) was used to prepare antimicrobial stock solutions.

### 3.3. Extraction of Essential Oils

The collected fresh basil leaves and stems were carefully washed and chopped into small pieces. The plant materials (35 kg) were subjected to steam distillation in a stainless-steel distillation system at an extraction temperature of 95–100 °C for 6 h. The collected oil-containing distillate was treated with anhydrous magnesium sulfate (MgSO_4_) to remove moisture, enhancing the oil’s purity and shelf life. The EOs were then transferred to tightly sealed amber glass vials and stored at 4 °C until further analysis.

### 3.4. Gas Chromatography–Mass Spectrometry (GC-MS)

The GC-MS analysis of the two basil EOs was performed using a SHIMADZU QP2010 Ultra GC-MS (Shimadzu, Kyoto, Japan), which featured SH-Rxi-5Sil MS capillary column (30 m × 0.25 mm, with a film thickness of 0.25 μm). A 0.5 μL sample of the EOs, diluted in hexane, was injected in split mode. The injector and transfer line temperatures were maintained at 320 °C and 200 °C, respectively. The oven temperature of the column started at 50 °C for 3 min, then increased gradually by 2 °C per minute until it reached 250 °C, where it was held for an additional 10 min. Helium served as the carrier gas, flowing at a constant rate of 1.19 mL/min. The mass spectrometer operated at an ion source temperature of 250 °C, in electronic impact (EI) mode with an energy of 70 eV, and capturing mass spectra in the range of 50–600 *m*/*z*. Identification of compounds was achieved by comparing the obtained mass spectra and retention indices to reference standards. A homologous series of alkanes (C5–C36) was analyzed under the same conditions to aid in calculating the retention index (RI). The constituents of the EOs were identified by comparing their mass spectra and retention indices with reference compounds from various libraries (NIST14.lib, NIST21.lib, and Wiley229.LIB). The relative abundance of each component was determined by calculating the percentage peak area in the total ion chromatogram without applying response factor corrections [117]. The GC-MS analysis was conducted three times, and the results are presented as the average peak area. Pure standards were purchased from Sigma-Aldrich (Steinheim, Germany) or Fisher Scientific (Pittsburgh, PA, USA).

### 3.5. Bacterial Growth Conditions and Cell Suspension Preparation

*L. monocytogenes* strain isolated from smoked salmon and sourced from the Ministry of Public Health, Qatar, was utilized in this study. The strain glycerol stock was preserved in microvials (Microbank^®^ microbial storage, Pro-Lab Diagnostics, Richmond Hill, ON, Canada) at −80 °C. For the experiments, the bacterial strain was adjusted to a turbidity corresponding to a 0.5 McFarland standard using phosphate-buffered solution (PBS, Atom Scientific, Manchester, UK) by inoculating a pure colony grown on Mueller-Hinton agar (MHA) plates (Liofilchem^®^, Roseto degli Abruzzi, Italy). The resulting culture was decimally diluted to achieve the desired concentration.

### 3.6. Antimicrobial Activity

The antibacterial activity of the EOs was evaluated by determining the minimum inhibitory concentration (MIC) in Mueller-Hinton Broth (MHB; VWR chemicals, Leuven, Belgium) against *L. monocytogenes* [10]. This was performed through a microdilution growth inhibition assay utilizing a Biotek Synergy LX Multi-Mode Reader (Biotek, Winooski, USA). In brief, 100 μL of double serial dilutions of the EOs (ranging from 40 to 0.156 mg/mL) were prepared in 96-well microdilution plates. Following this, 100 μL of each bacterial strain was added to the wells. The same procedure was applied to test individual active components (linalool, eugenol, estragole, and eucalyptol) against the *L. monocytogenes* strain. No bacteria were added to the negative control wells, while DMSO without any antimicrobial agents served as the positive control. The plates were incubated at 37 °C with continuous shaking, and optical density (OD) was measured at 600 nm. The MIC was defined as the lowest concentration of the antimicrobial agent required to inhibit visible bacterial growth in microdilution wells after incubation.

### 3.7. Total Phenolic Content

Total phenolic content (TPC) of the two *Ocimum* EOs was determined using the Folin–Ciocalteu colorimetric oxidation/reduction-based reaction as previously described [118]. 15 µL of extracts that had been diluted 10 times with methanol was added to 80 µL of Folin–Ciocalteu reagent (diluted 10 times with distilled water). After waiting 5 min, 100 µL of 5% saturated sodium carbonate solution was added. The reaction oxidation products exhibit a broad blue light absorption spectrum with a maximum at 760 nm. OD 760 for the different mixtures was measured 30 min later against a blank using a spectrophotometer. A calibration curve was prepared using a strong antioxidant, gallic acid, and the results were expressed as gallic acid equivalents (GAEs) in micrograms per gram of the EO.

### 3.8. Antioxidant Activity Assessment

The antioxidant potential of the *Ocimum* EOs and the selected active components (linalool, eucalyptol, eugenol, and estragole) was evaluated using the protocol of Brand-Williams et al. [119].An equivalent volume of a 0.5 mM solution of 2,2-Diphenyl-1-picrylhydrazyl (DPPH; ref D9132, Sigma-Aldrich, St. Louis, MO, USA) in methanol was mixed with various concentrations of *Ocimum* EOs, eugenol (at 1.25, 2.5, 5, 10, and 50 μg/mL), linalool, eucalyptol or estragole (at 100, 200, 300, 400, 500, 800, and 1000 μg/mL). After allowing the mixture to react for half an hour at room temperature in the dark, absorbance at 517 nm was measured using a spectrophotometer. Ascorbic acid was used as positive control and methanol was used as a blank. The percentage of radical scavenging activity was calculated using the formula below.

$$\%\;\mathrm{Radical}\;\mathrm{scavenging}\;\mathrm{activity}=\left(\frac{OD\;blank-OD\;plant\;essential\;oil\;at\;each\;concentration}{\begin{array}{c} \mathrm{OD}\;\mathrm{blank}\; \end{array}}\right)\times 100$$

The percentage radical scavenging activity was plotted against the concentration of plant EOs or active components, and the plotted curves were utilized to establish the half-maximal effective concentration (EC_50_) of DPPH free radical inhibition. EC_50_ values were estimated by nonlinear regression using GraphPad Prism 10.0 (GraphPad Software, LLC, Boston, MA, USA) with a four-parameter logistic model and variable slope. Model fit was assessed, and 95% confidence intervals were calculated for EC_50_ estimates. For linalool, estragole, and eucalyptol, EC_50_ values exceeding 1000 μg/mL were extrapolated from the fitted dose–response curves. For comparison, the EC_50_ of the reference antioxidant L-ascorbic acid was determined.

### 3.9. Antibiofilm Assessment Against L. monocytogenes

#### 3.9.1. Biofilm Formation on Stainless Steel Surfaces

Circular stainless steel (SS) coupons (304 L, B.M.T, Beirut, Lebanon), measuring 41 mm in diameter and 1 mm thick, were used as surfaces for biofilm formation. Prior to use, the coupons were prepared and sterilized according to the method outlined by Mechmechani et al. [10]. Planktonic bacterial suspensions of *L. monocytogenes* at a concentration of 10^7^ CFU/mL were prepared in PBS and applied to the sterilized stainless-steel coupons. The coupons were incubated at room temperature for 1 h to enhance bacterial adhesion. After incubation, the 5 mL of suspension was discarded, and the coupons were thoroughly washed with PBS to remove any non-adhering cells. The slides containing adherent cells were then covered with 5 mL of Tryptic Soy Broth (TSB, Liofilchem^®^, Roseto degli Abruzzi, Italy) medium and incubated for 24 h at 37 °C. Following incubation, the TSB medium was discarded, and the biofilms were rinsed with PBS to eliminate remaining planktonic cells. The rinsed slides were subsequently used for antibiofilm treatments and to quantify biofilm-associated viable cells.

#### 3.9.2. Biofilm Removal

To evaluate antibiofilm efficacy against *L. monocytogenes*, established biofilms were exposed to 3 mL of OB-S EO or selected active components (linalool, eugenol, estragole and eucalyptol) at their correspondent ½ MIC, 1 MIC and 2 MIC values and incubated at room temperature for 15 min. Following treatment, the slides were immersed in 5 mL of Dey–Engley neutralizing solution to stop the residual antimicrobial activity of the essential oils and their major constituents, thereby preventing any carryover effect during serial dilution and enumeration of surviving bacterial cells [120]. Control biofilms were treated with phosphate buffer containing DMSO without any antimicrobials. For quantifying biofilm-associated viable cells, the slides were transferred into 20 mL of PBS using a sterile 100 mL pot. Attached cells were detached by vortexing at maximum speed for 2 min. Serial dilutions were then prepared in PBS and plated onto Tryptic Soy Agar (TSA; Liofilchem^®^, Roseto degli Abruzzi, Italy) plates. After incubating the plates for 24 h at 37 °C, the number of cells was counted, and the results were expressed as log CFU/cm^2^. The data represents the average from two independent experiments.

### 3.10. Statistical Analysis

All treatments were performed in duplicate, with two replicates for antibiofilm assessments. The statistical significance of the results was evaluated using GraphPad Prism 10.0 software. One-way ANOVA was applied, followed by Tukey’s multiple comparison tests, to identify differences in mean among the treatment groups. A *p*-value of less than 0.05 was considered statistically significant.

## 4. Conclusions

This study demonstrates that two *Ocimum* species cultivated under the arid environmental conditions of Qatar possess distinct chemotypic compositions associated with markedly different biological activities. The linalool-rich *O. basilicum* EO exhibited enhanced antimicrobial and antioxidant activities compared with the citral-rich *O. africanum* EO, highlighting the influence of chemotypic variation on functional bioactivity. While the whole *O. basilicum* EO showed the greatest antimicrobial efficacy against planktonic *L. monocytogenes*, evaluation of its individual constituents revealed that selected phytocompounds, particularly eugenol, displayed substantially greater antibiofilm activity against established *L. monocytogenes* biofilms following a short 15 min exposure. These findings suggest that these natural agents may have broader antimicrobial and antibiofilm potential. Future investigations could evaluate their efficacy against a wider range of clinically relevant pathogens, thereby extending their application beyond food safety into the medical field, where biofilm-associated infections and antimicrobial resistance (AMR) remain major challenges. Additionally, their antioxidant capacity suggests applications in pharmaceuticals, nutraceuticals, and bioactive coatings for medical implants to assist infection control. Industrially, future research should prioritize real-world applications. Prospective applications of these bioactive compounds include their use in alternative Clean-in-Place (CIP) systems, functional antioxidant preservation, and active biodegradable food packaging to improve food safety, extend shelf life, and meet clean-label consumer demands. However, to successfully transition these natural solutions to the market and satisfy consumer clean-label demands, comprehensive safety assessments are required, particularly for constituents such as estragole. Furthermore, all products must obtain the necessary regulatory approval before application in commercial food-preservation or -packaging systems.

## Figures and Tables

**Table 1:** Chemical composition of the essential oils of *Ocimum basilicum* L. (OB-S) and *Ocimum africanum* Lour. (OA-L).

No.	RT (min)	RI_Lit_	RI_Calc_	Compound Identification	Area % (OB-S)	Area % (OA-L)
1	4.946–4.949	845	844	4-Hydroxy-4-methylpentan-2-one (β-hydroxy ketone) ^[b]^	**7.47**	**10.92**
2	8.422–8.426	948	949	α-Pinene ^[a]^	0.92	–
3	10.354	897	895	Sabinene ^[a]^	0.87	–
4	10.547–10.554	943	944	β-Pinene ^[a]^	1.95	–
5	11.332–11.343	958	957	β-Myrcene ^[a]^	0.60	–
6	13.611	1059	1057	1,8-Cineole ^[a]^	**12.91**	–
7	14.676–14.683	976	975	β-Ocimene ^[b]^	2.01	–
8	18.075–18.352	1082	1080	Linalool ^[a]^	**31.53**	**1.92**
9	20.733	1121	1120	Camphor ^[a]^	–	–
10	22.522	1138	1137	*endo*-Borneol ^[a]^	0.99	–
11	24.383–24.396	1172	1170	4-Allylanisole ^[b]^	1.82	–
12	25.632–30.023	1277	1277	Bornyl acetate ^[b]^	1.70	–
13	27.156	1174	1174	Neral/(Z)-Citral ^[a]^	–	**33.01**
14	29.204	-	1176	Geranial/(*E*)-Citral ^[a]^	–	**45.33**
15	34.555	1392	1390	Eugenol ^[a]^	3.17	–
16	36.441	1352	1353	Geranyl acetate ^[a]^	–	1.22
17	36.513	1267	1266	(Z)-Methyl cinnamate ^[a]^	–	–
18	36.815	1398	1398	β-Elemene ^[b]^	2.88	–
19	38. 812	1494	1493	β-Caryophyllene ^[a]^	–	1.24
20	39.584–43.636	1490	1491	α-Bulnesene ^[b]^	3.39	0.56
21	39.601–39.602	1430	1428	α-Bergamotene ^[b]^	**6.11**	–
22	42.276–42.279	1515	1515	Germacrene D ^[b]^	**6.42**	–
23	43.162	1431	1431	γ-Elemene ^[a]^	1.38	–
24	43.810	1503	1500	Germacrene A ^[b]^	2.57	–
25	44.285–44.289	1435	1436	γ-Cadinene ^[a]^	3.86	–
26	48.122	-	1499	Caryophyllene oxide ^[a]^	–	**2.74**
27	51.741–51.777	1580	1581	τ-Cadinol ^[b]^	3.91	–

RT = Retention time; RILit = Retention index in the literature; RICalc = Retention index relative to alkanes (C5–C36); compound identified by comparison of the mass spectrum with the NIST14.lib and WILEY229.LIB spectral libraries; ^[a]^ compound identified by comparison with pure standard; ^[b]^ compound identified by interpretation of the mass spectrum fragmentation pattern according to data described in the literature. Bolded compounds represent the five most abundant constituents (highest relative area %) in samples OB-S and OA-L. Underlined compounds indicate constituents detected in both OB-S and OA-L samples.

**Table 2:** Minimum inhibitory concentrations (MIC in mg/mL) of basil essential oils and selected active components against *L. monocytogenes* strains.

Essential Oils or Active Components	*Listeria monocytogenes* MIC (mg/mL)
OB-S	5
OA-L	10
Eugenol	10
Linalool	20
Estragole	40
Eucalyptol	40

OB-S: *Ocimum basilicum* L., OA-L: *Ocimum africanum* Lour.

**Table 3:** DPPH radical scavenging activity (EC50) of basil essential oils and selected active components.

Essential Oils or Active Components	EC50 (µg/mL)
OB-S	162.69 ± 0.78
OA-L	939.38 ± 0.68
Ascorbic acid	23.91 ± 0.3
Eugenol	5.31 ± 0.67
Linalool	>1000 (extrapolated EC_50_ = 3655.3 ± 2.01) ^a^
Estragole	>1000 (extrapolated EC_50_ = 4860.7 ± 1.19) ^a^
Eucalyptol	>1000 (extrapolated EC_50_ = 4974.09 ± 1.55) ^a^

OB-S: *Ocimum basilicum*, OA-L: *Ocimum africanum* Lour. EC50: Half-maximal effective concentration (the concentration required to achieve 50% radical scavenging activity). Values are mean ± SD. ^a^ EC_50_ values > 1000 µg/mL are extrapolated from the fitted dose–response curves.

## Data Availability

All relevant data are included within this manuscript.

## Abbreviations

The following abbreviations are used in this manuscript:

OB-S	*Ocimum basilicum* L.—sweet basil
OA-L	*Ocimum africanum* Lour.—lemon basil
